# Supplementary material for: An optical method for deriving the anterior and posterior curvatures of intraocular lenses using dual back-vertex power measurements
Source: PLoS One. 2025 Dec 2;20(12):e0337041. doi: 10.1371/journal.pone.0337041 (PMC12671823; doi:10.1371/journal.pone.0337041)
Supplement: Attachment — Submitted filename: An_optical_method_for_deriving__Gatinel.pdf [file pone.0337041.s001.pdf]

Review on: An optical method for deriving the anterior and posterior curvatures of IOLs using dual back-vertex power meausrements (PONE D-25-24939)

Implantation of IOLs need information on the optical power of the lens. Beyond that geometrical information on the shape of the lens are useful. Because manufactures seldom provide "construction data", the author proposes a method to retrieve at least the curvatures of the surfaces from the refractive index, the lens thickness, the power of the lens and two additional measurements. Since back vertex power measurements are handy, the author relies on this approach. He includes a technical detail regarding an offset, denoted by d, due to the haptic angulation.

The manuscript contains valuable findings. However, there is room to shorten and condense the manuscript.

Here follow my comments and suggestions:

- The final result (eq. 8) is derived in terms of vergences in appendix S1. A much easier and shorter derivation, based on paraxial ray data, is included at the end of this review. Therefore, cancel eq. (7) and S1.
- The Python code might be distributed on request by the author, but should not be included in the article.
- The change in back-vertex power by the offset d can be compensated for by an "upstream" transfer of the vertex, as is implicitly included in eq. 6. However I don't see, why the lengthy equation (6) is needed here. In a numerical calculation, one just corrects for d, leading to say $A^*(d)$ and $B^*(d)$, which enter the result in eq. 8. Therefore, cancel eq. (6).
- The Results section contains interesting information, mainly presented in tables Tab. 1 and Tab. 2. Personally, I would prefer a graphical presentation, which would be much easier to grasp.
- The paragraph between line 152 and 169 is hard to understand, because it refers to material outside the scope of the manuscript. Could the author elaborate on this topic or leave it outside?
- The section on assumptions and limitations mentions higher order aberrations, but does not include lower aberrations depending on the form of the IOL or on the tilt / decentration of an IOL. A quite short note might be helpful.

Derivation of eq. 8

We use the paraxial so-called $y - nu$ Method, which treats ray heights y and ray angles u. For 2 surfaces with powers P_1 and P_2, separated by the reduced

distance $\tau = t/n_{IOL}$ we have the following raytrace equations

$$n_{IOL}u_1 = -y_1 P_1 \quad (1)$$
$$y_2 = y_1 + \tau n_{IOL} u_1 \quad (2)$$
$$u_2 = n_{IOL}u_1 - y_2 P_2 \quad (3)$$
$$l_2' = 1/B = -y_2/u_2 \quad (4)$$

where the ray height on surface 1 is chosen as $y_1 = 1$ and the refractive index outside the lens is set to 1.

Eq. (4) and (2) lead to

$$u_2 = -(1 + \tau n_{IOL} u_1)B$$

Using Eq. (1) results in

$$u_2 = -(1 - \tau P_1)B$$

Implementing eq. (3) renders

$$-(1 - \tau P_1)B = -P_1 - P_2 + \tau P_1 P_1$$

Introducing the equivalent power $P = P_1 + P_2 - \tau P_1 P_1$ we finally arrive at

$$P_1 = \frac{1}{\tau}(1 - P/B)$$

By considering the reverse situation, i.e. flipping indices $1 \leftrightarrow 1$ and $B \rightarrow A$, we get without further calculation

$$P_2 = \frac{1}{\tau}(1 - P/A)$$

This completes eq. 8.
