# Supplementary material for: An optical method for deriving the anterior and posterior curvatures of intraocular lenses using dual back-vertex power measurements
Source: PLoS One. 2025 Dec 2;20(12):e0337041. doi: 10.1371/journal.pone.0337041 (PMC12671823; doi:10.1371/journal.pone.0337041)
Supplement: Attachment — Submitted filename: Response_to_Reviewers.docx [file pone.0337041.s002.docx]

**Response to Reviewers**

*Manuscript: An optical method for deriving the anterior and posterior curvatures of IOLs using dual back-vertex power measurements*

Corresponding author: D. Gatinel

We thank the reviewers for their careful reading and constructive suggestions. Below we address each point in sequence and indicate exactly what we changed and where it appears in the revised manuscript (section, equation, table, or figure label). Where relevant, we reproduce key formulae for clarity.

**Global editorial change (resubmission).**

Following the reviewers' and editor's guidance, we have removed the supplementary the (now removed) appendix. No cross-references to S2 remain in the revised manuscript. For transparency, the minimal script used to assemble the summary tables/figures will be made available upon reasonable request to the corresponding author; it is not included as an appendix.

**Reviewer #1**

**Point 1 — Cancel Eq. (7) and Appendix S1 and replace with a shorter paraxial derivation.**

Action taken. We removed the previous Eq. (7) and Appendix S1 and placed a concise paraxial y–ν derivation directly in the Methods under Back‑Vertex Power and Analytical Solution (d = 0). The derivation leads to the relations used in the paper and avoids the long appendix.

**Point 2 — Do not include the Python code in the paper.**

Action taken. The code appendix has been removed. We now state that the internal script used to generate tables and figures is available on request. This note appears adjacent to Table 1 and Table 2.

**Point 3 — Handle the axial offset d via an upstream vertex transfer; cancel Eq. (6).**

Action taken. We deleted the former Eq. (6) and now correct measured bench readings taken at spacing d using the standard paraxial propagation identity and its inversion (both stated once in Methods under Offset correction for a measurement spacing (d ≠ 0)):

We apply the inversion to map raw bench readings to vertex‑plane powers before using the d = 0 solution:

(See Eq. (10) and Eq. (11) in the revised manuscript.)

**Point 4 — Prefer a graphical presentation of the Results in addition to tables.**

Action taken. We retained the tables and added two figures that plot the same datasets for immediate visual interpretation:

- Fig. 2: effect of axial spacing d on the recovered radii; plotted from Table 1.
- Fig. 3: effect of thickness t on the recovered radii at d = 0; plotted from Table 2.

**Point 5 — The paragraph between lines 152–169 was hard to understand; either elaborate or remove.**

Action taken. We replaced the prior paragraph with a concise, in‑scope explanation located in Results (transition to the sensitivity analysis). The new text now present in the manuscript reads:

Knowledge of the IOL design is particularly critical for high‑power implants, as the labeled power is typically defined relative to the image‑side principal plane of the implant. For a biconvex IOL, this principal plane is located between the anterior surface (in a nearly convex‑plan configuration) and the posterior surface (in a nearly plan‑convex configuration). In low‑power implants, the uncertainty associated with the principal plane position is more constrained due to their thinner profile, as demonstrated by Gatinel et al. (2022) in a theoretical thick lens model [10]. The quadratic dependence on IOL power underscores that the impact of ELP variations on postoperative refraction increases significantly with higher IOL powers, making the IOL design a more critical factor for accurate power calculations in such cases [11]. Accurate thick‑lens parameters are most impactful when lens power and thickness are high, because the location of the principal planes and ELP‑to‑refraction coupling increase with P. Our contribution is limited to retrieving (P1, P2) (and thus the Coddington shape) from dual back‑vertex measurements; how this information should be incorporated into a given formula or ray‑tracing pipeline is outside our scope.

This replacement keeps the discussion focused and directly motivates the two worked subsections Case 1: d = 0 and Case 2: Sensitivity to d ≠ 0 and t.

**Point 6 — Add a short note on lower‑order aberrations and on tilt/decentration.**

Action taken. We added Lower‑order aberrations and alignment to Assumptions and Limitations, explaining how modest tilt/decentration and form‑dependent lower‑order aberrations can bias bench readings if not well controlled.

**Reviewer #2**

**Point 1 — Figure 1 is too schematic (beams, BVP meaning, indices, labels A/B, angle α); also cite ISO 11979‑2.**

Action taken. We redesigned Fig. 1 and updated the Methods/Setup text:

- The caption now explicitly shows a collimated input and a convergent output beam for normal and flipped orientations, and clarifies that A and B are back‑vertex powers (not distances).
- The refractive indices n_a (ambient) and n_IOL (optic) are indicated in the schematic and caption.
- The two configurations are labeled (A) and (B) in both drawing and caption.
- We added a panel depicting a finite spacing d linked to haptic angulation α, and we show how these readings are corrected back to the vertex plane before analysis (see Eq. (11)).
- The Methods/Setup now cites the ISO 11979‑2 framework for back‑vertex power metrology and alignment.

**Point 2 — Support the 5°–15° haptic angle range with a reference.**

Action taken. We added a clinical reference supporting typical haptic angulation and IOL positional behavior (Petternel et al., 2004, included in the revised references) and made the geometric implication explicit in Assumptions and Limitations:

We incorporate this offset d via the vertex‑transfer correction (Eq. (11)). Numerically solving the corrected system yields the same final outputs (P, P1, P2).

**Point 3 — Unify nomenclature for back‑vertex power across d = 0 and d ≠ 0.**

Action taken. We introduced a dedicated Nomenclature and wavelength paragraph that (i) defines A ≡ P_BV^(normal)(d = 0) and B ≡ P_BV^(flipped)(d = 0), (ii) denotes bench readings at finite spacing as A_meas(d) and B_meas(d), and (iii) maps them back to the vertex plane using Eq. (11) before applying the d = 0 relations.

**Point 4 — Augment Table 1’s caption with the actual P, A, B, t for the low/medium/high examples.**

Action taken. The captions of Table 1 and Table 2 now list the exact (A, B, t) used in each exemplar and indicate the resulting intrinsic power P (with consistent values across the tables and new figures).

**Point 5 — Address the wavelength used for BVP metrology and the dispersion of n_IOL and n_a.**

Action taken. In Nomenclature and wavelength we state that all vergences and indices refer to the bench wavelength λ (noting the common Hg e‑line at 546.07 nm). We emphasize using n_IOL(λ) and n_a(λ) at the same λ as the BVP measurement. Because recovered radii scale with (n_IOL − n_a)/P1,2, even small wavelength mismatches can bias R1,2, which motivates consistent dispersion data.

**Point 6 — Discuss the impact of uncertainty in n_IOL (ISO specifies to the third decimal).**

Action taken. We added a brief sensitivity note in Assumptions and Limitations describing how ±0.001 uncertainty in n_IOL can shift R1 and R2, especially for higher powers, and how using material dispersion at the bench wavelength mitigates this source of bias.

**Point 7 — Consider Fernández et al. (2025) in the Discussion.**

Action taken. We added the study and expanded the Discussion accordingly. The new text inserted into the manuscript reads:

Recent clinical work in multifocal IOLs reported that, with optimized constants, a thick‑lens formula showed no clinically relevant prediction‑error advantage over Barrett in the 18–27 D range, and using exact versus labeled powers did not materially change accuracy in that cohort [12]. However, even if such constant adjustment can effectively zeroize the mean prediction error across a population, this approach does not prevent potentially large individual errors, particularly in high‑power lenses where design‑related variability has a stronger clinical impact. In this context, our method is complementary: by recovering (P1, P2) and the shape factor from dual back‑vertex measurements, it provides geometry that can help mitigate such individual risks.

**Additional notes on equations and where to find them**

- Back‑vertex power correction for spacing d (Methods, Offset correction):

- Analytical relations used after d‑correction (Methods, d = 0 case):

where A and B are vertex‑plane back‑vertex powers (normal/flipped).

- Figures and Tables (Results): Fig. 1 (re‑designed schematic with beams, indices, labels, and d); Fig. 2 and Fig. 3 (graphical trends); Table 1 and Table 2 (explicit (A, B, t) and P reported in captions).
- Worked examples and structure (Results): Case 1: d = 0 (worked numerical example; see Eq. (12)) and Case 2: Sensitivity to d ≠ 0 and t.
- Terminology and dispersion: Nomenclature and wavelength paragraph (unified symbols; indices and vergences referenced to the bench wavelength).
- Alignment note: Assumptions and Limitations, subsection Lower‑order aberrations and alignment.

**Summary of revisions**

- Removed Eq. (7) and Appendix S1; added a compact in‑text paraxial derivation (Methods).
- Deleted former Eq. (6); implemented vertex‑plane correction for finite spacing d (Methods, Eq. (10), Eq. (11)).
- Unified BVP nomenclature and added wavelength/dispersion guidance (Nomenclature and wavelength).
- Re‑designed Fig. 1 with beams, indices, labels, and d; cited ISO 11979‑2 in Methods.
- Added Fig. 2 and Fig. 3; expanded table captions with explicit (A, B, t) and P.
- Replaced the previously unclear paragraph with a focused, in‑scope explanation (Results).
- Added a note on lower‑order aberrations and alignment (Assumptions and Limitations).
- Removed code appendix; code available on request.
- Added and discussed Fernández et al. (2025) in the Discussion.
- Added a clinical reference supporting typical haptic angulation and quantified its geometric implication.

We appreciate the reviewers’ input; these changes improved clarity, rigor, and practical utility for both clinicians and optical scientists.
